# Exploring delayed diagnosis in Gaucher disease: insights from a community survey and potential solutions

**DOI:** 10.1186/s13023-026-04209-5

**Published:** 2026-02-20

**Authors:** Diana Paulina Peña Aragón, Tanya Collin-Histed, Magy Abdelwahab, Jesús Villarrubia Espinosa, Raul Chertkoff, Andre Balzekiene, Jessica Pacey

**Affiliations:** 1 International Gaucher Alliance, Cuernavaca, Mexico; 2 International Gaucher Alliance, London, UK; 3 Cairo University Pediatric Hospital, Kasr Alainy Hospital, Cairo, Egypt; 4 University Hospital Ramón y Cajal, Madrid, Spain; 5 International Gaucher Alliance, Tel Aviv, Israel; 6 International Gaucher Alliance, Vilnius, Lithuania; 7 67health, London, UK

**Keywords:** Gaucher disease, Diagnosis, Survey, Awareness

## Abstract

**Background:**

Gaucher disease is a rare lysosomal storage disorder caused by insufficient activity of the enzyme β-glucocerebrosidase. This leads to the accumulation of fatty deposits in cells and tissues and damages multiple organ systems. Diagnosing Gaucher disease often involves a prolonged and challenging “diagnostic odyssey”. The International Gaucher Alliance (IGA) conducted a survey among individuals living with Gaucher disease, including patients, families, and caregivers, to investigate diagnostic challenges. Distributed primarily in English language, with a Spanish translation for relevant IGA members, the survey was completed by 142 respondents from 40 different countries between November 2024 and February 2025. The study aims to guide future efforts to promote timely diagnosis, access to expert management, and treatment before irreversible damage occurs. Early diagnosis also brings emotional and practical benefits, including informed family planning and support networks.

**Results:**

The survey revealed that diagnostic delays remain prevalent, with 58% of respondents waiting more than a year for diagnosis. Key contributors to delays included low awareness among physicians and inadequate communication between medical specialties. Common “red flag” symptoms identified in guidelines were often linked to eventual diagnosis, highlighting the need to educate healthcare professionals, especially those likely to encounter patients but not consider Gaucher disease (e.g., internal medicine specialists and gastroenterologists). Notably, 17% of respondents were not made aware of the genetic nature of Gaucher disease when they were diagnosed, potentially missing opportunities to identify affected relatives or influence family planning. Respondents suggested that increased awareness among healthcare professionals, expanded access to newborn screening, and greater availability of genetic and enzymatic testing could significantly accelerate the process.

**Conclusions:**

As well as continuing to engage in education and awareness activities for specialists that commonly diagnose Gaucher, targeted awareness campaigns for secondary care clinicians that do not commonly diagnose Gaucher, but are regularly seen in the journey to diagnosis could reduce diagnostic delays. Providing lay-friendly resources to help specialists explain genetic inheritance to newly diagnosed patients may enhance early family diagnoses. Country-specific surveys to understand local diagnostic experiences could shape tailored interventions. Lastly, advocacy efforts to address access barriers, should amplify patient voices and prioritise community needs.

**Supplementary Information:**

The online version contains supplementary material available at 10.1186/s13023-026-04209-5.

## Background

Gaucher disease is a rare lysosomal storage disorder characterised by insufficient activity of the enzyme β-glucocerebrosidase, resulting in accumulation of glucocerebroside and other glycolipids (build-up of fatty deposits) in the lysosomes of cells and tissues which cause damage to multiple organ systems, primarily affecting the liver, spleen and bones [1, 2]. There are two main classifications; non-neuronopathic (or type 1) and neuronopathic (or types 2 and 3) and so patients present with a range of symptoms and there is a continuum of disease severity (phenotype) [3]. 

Gaucher disease is rare, with an incidence of 1 case per 40,000–60,000 births in the general population, but this can rise to 1 per 800 in the Ashkenazi Jewish population, with type 1 being the most common form, accounting for 90–95% of Gaucher disease [2]. 

Diagnosis is challenging, as in many rare diseases, due to limited disease awareness among clinicians, who prioritise investigation of other diagnostic possibilities with higher mortality or with available treatment [3, 4]. Diagnosis is also confounded by the non-specific and heterogeneous nature of Gaucher disease symptoms, which can include fatigue, thrombocytopaenia, recurrent wheezy chest or chest infections, and hepatosplenomegaly, as well as bone pathologies [5]. 

For all these reasons, diagnosis of Gaucher takes a long time, often referred to as a “diagnostic odyssey” that is familiar to those working in rare disease [4], and delays of up to 20 years before confirmation of diagnosis from first symptoms have been reported anecdotally [6]. 

The International Gaucher Alliance (IGA) and medical experts in the field recognise that timely diagnosis is essential so that appropriate care and therapy can be initiated before build-up of damaging fatty deposits and associated, potentially irreversible, symptoms [7]. 

Extensive work has been done in recent years to identify common “red flags” that point towards a diagnosis of Gaucher disease and these are now clearly outlined in the literature [1, 3, 5]. Detailed diagnostic guidelines have also been developed and are readily available [1, 3, 8]. Moreover, the diagnostic tests to confirm a diagnosis are well-known and are well established [9, 10]. However, it appears that access to these tests is typically restricted to specialist centres, where they exist, to confirm patient clinical presentation, exact mutation and sometimes, a likely prognosis. Where there is no funded or approved treatment for Gaucher disease, there may be less willingness to pay for diagnostic testing, despite the benefits to the individual of knowing if they have the disease.

The IGA is a patient-led international organisation that represents over 90% of the Gaucher community and has built its reputation through listening to and delivering outcomes that have impacted patients’ and their carers’ lives. Its vision is a world where all Gaucher patients have access to the treatment and care they need and there is the possibility of a cure.

A select working group of the IGA, comprising both community and clinical experts, including a representative of the scientific body, the International Working Group on Gaucher Disease (IWGGD), agreed to conduct a survey of people living with Gaucher disease (patients as well as their families and caregivers) to explore the challenges of diagnosis in detail. Specifically, the IGA sought to uncover any insights that could reduce the time to diagnosis by understanding how many and which different specialists a person with Gaucher disease typically sees before receiving their diagnosis, identifying the main specialisms involved in the eventual diagnosis and understanding any wider challenges to the process from non-clinical perspectives. The aim is to use the findings to direct future collaboration and activities to raise awareness of Gaucher disease, support timely diagnosis and access to expert management and treatment before the build-up of fatty deposits in the organs becomes damaging and, in some cases, irreversible. Early diagnosis has many benefits for families beyond treatment and multidisciplinary care, including connecting with other families and patient groups for emotional and practical support, as well as to make informed decisions regarding family planning.

Here we will discuss in detail how the survey was undertaken, with consolidated findings and recommendations to support diagnosis for people living with this demanding, chronic condition, based on outputs of the survey.

## Methods

### Research channel selection

An online survey was conducted to understand the diagnostic process for Gaucher disease from the patient perspective so we can identify potential ways that might expedite diagnosis and speed up access to appropriate management and care. The IGA has over 58 member associations, representing 54 countries throughout the world, comprising patients, caregivers, physicians and affiliated healthcare practitioners. A survey disseminated via the IGA members would allow greater understanding of issues around diagnosis, although the bias towards well-informed patients and caregivers is recognised.

### Project oversight

Oversight of the survey project was the responsibility of a dedicated working group of the IGA, who identified priority areas for patient questioning. Specialist support was provided to design and implement the survey by 67health, a UK-based healthcare communications organisation. Initial literature reviews were undertaken to establish the scientific and clinical knowledge base, allowing the survey questions to focus on gaps in understanding where detailed patient insight would be of most value.

### Online survey implementation

SurveyMonkey was selected as the most appropriate tool: simple, efficient and easy for respondents to access. Question topics were divided into three sections: demographics, diagnostic journey and communication; and the full questionnaire can be found in the additional files accompanying this report.

The survey was designed with a mix of tick box / multiple choice responses alongside open text questions to ensure that the nuance of feedback would be clear, particularly for responses around communication and terminology used. All respondents consented to participate in the survey, and responses were anonymised, although the option was given to participate in 1:1 interview style follow up sessions if needed. No 1:1 sessions have been undertaken to date, so all data presented here is based on the online survey responses only.

The survey was reviewed by the working group and piloted prior to distribution to members of the IGA at the annual meeting in November 2024. The survey was primarily distributed in English language, with a Spanish translation that was distributed to Spanish speaking IGA members. Responses were collated from November 2024 to February 2025.

### Analysis

Once responses were received, the data were analysed in Microsoft Excel and using sentiment analysis (via an online tool, Perplexity AI, which uses a large language model to determine whether the respondent’s attitude is positive, negative, or neutral). The use of AI to analyse the sentiment of open text questions alleviated capacity and resource requirements for the IGA, without being hypothesis generating, which was all conducted by humans. Based on these findings, the working group was convened to review the findings and draw conclusions and recommendations from the survey.

## Results

**Fig. 1:**
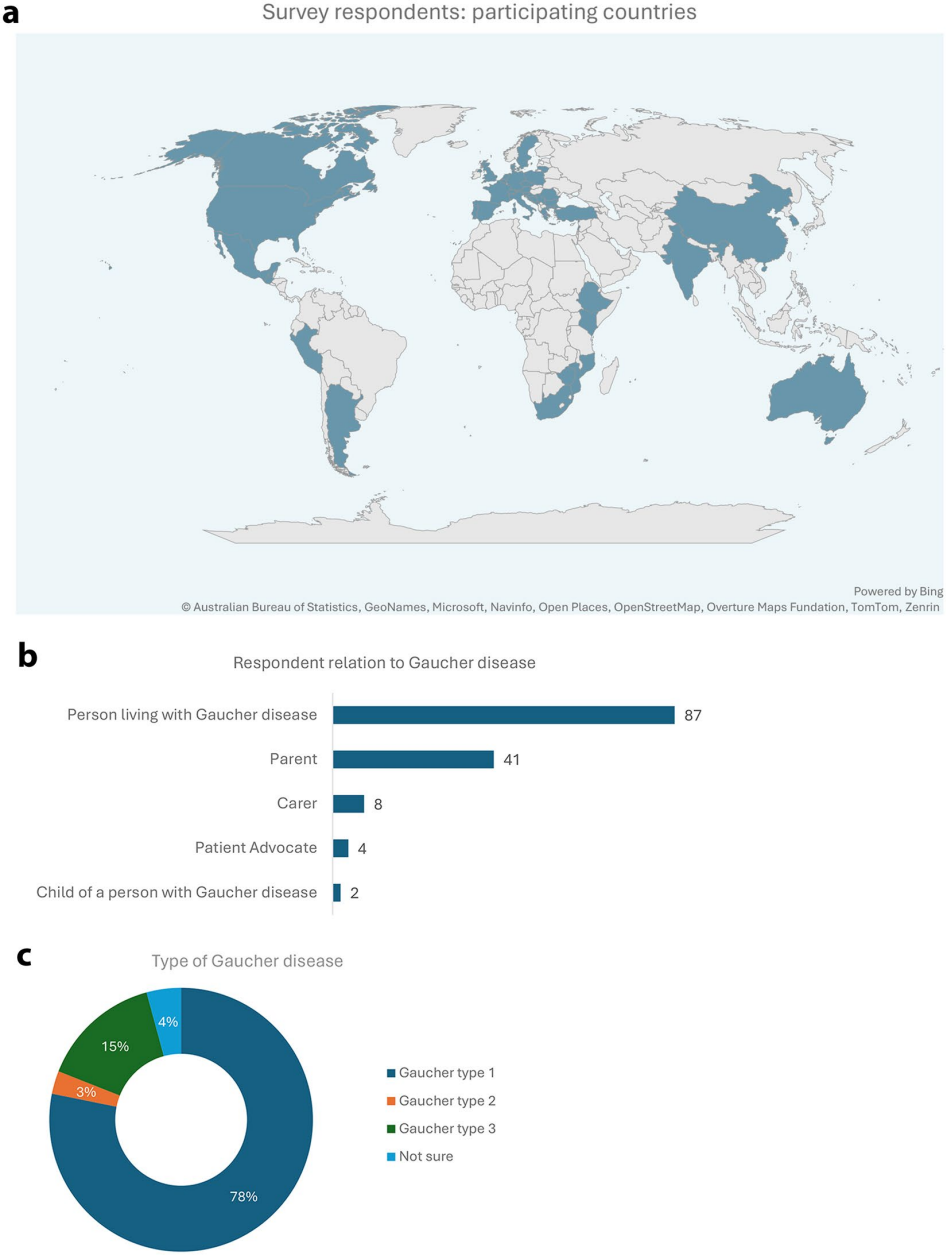
Respondent demographics: **a**) Countries from which the respondents to the survey are located, as shown on a map; **b**) The total number of respondents and their relation to Gaucher disease; **c**) The distribution of the type of Gaucher disease among all respondents

A total of 142 respondents completed the survey from 40 different countries. Figure 1 (see components 1a, 1b and 1c) shows the demographics of the 142 people living with Gaucher disease or their caregivers who responded to the survey online.

**Fig. 2:**
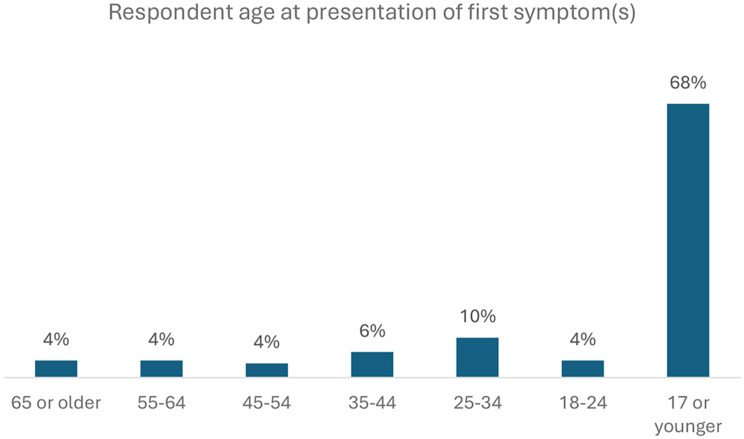
The age when respondents or their family / carer first experienced symptoms associated with Gaucher disease

**Fig. 3:**
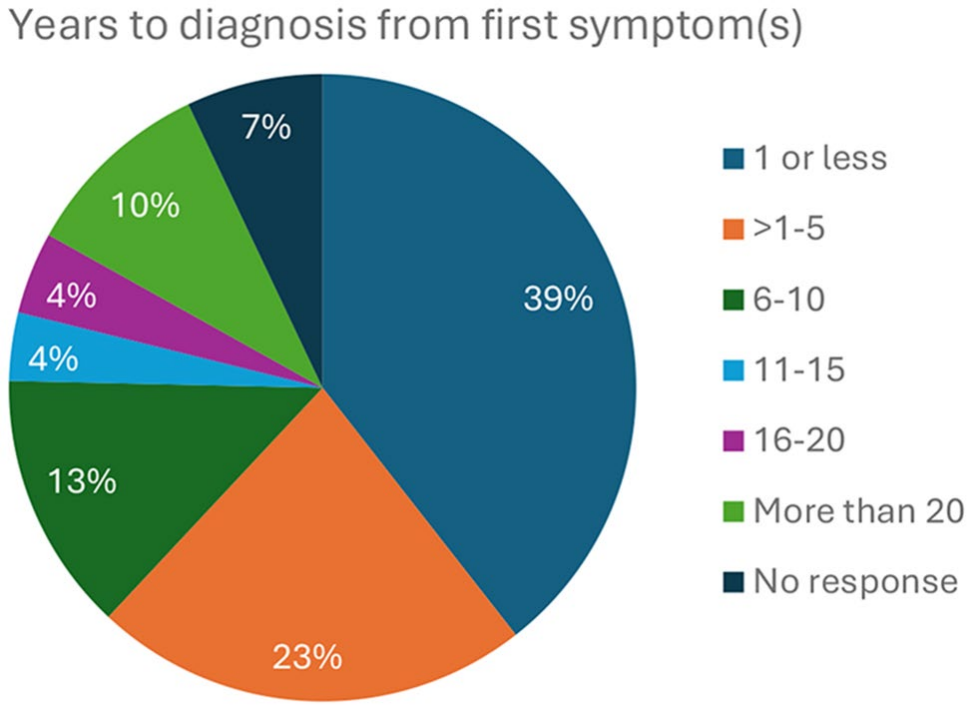
The time to confirmed diagnosis after first symptoms as estimated by respondents to the survey

The age of presentation of symptoms suggestive of Gaucher disease and time lapse between presentation and diagnosis is presented below. The majority of respondents (68%) experienced their first symptoms in childhood (before the age of 18) (Fig. 2). The time between initial symptoms and confirmation of a diagnosis of Gaucher disease varied from 1 year or less (39%) to more than 20 years (10%) (Fig. 3). Among respondents only, 58% (76 out of 132) experienced more than a year delay to diagnosis.

When asked the question: “How satisfied were you with the process of diagnosis of Gaucher disease?”, respondents could mark their degree of satisfaction on a linear scale, and responses varied from 0 (completely dissatisfied) through to 100 (completely satisfied), with a mean of 63.

Respondents had the opportunity to add a supplementary comment. Sentiment analysis showed that, overall, 50% of responses provided were negative expressing frustration, pain, and dissatisfaction with the diagnostic process, particularly regarding delays, invasive procedures, lack of knowledge among doctors, and misdiagnoses. Thirty percent (30%) of responses were neutral giving factual accounts of the diagnostic process without strong positive or negative emotions, acknowledging some improvements over time. Twenty percent (20%) of responses were positive and express gratitude for relatively quick diagnoses, satisfaction with current treatment, or relief at finally understanding their condition.

**Fig. 4:**
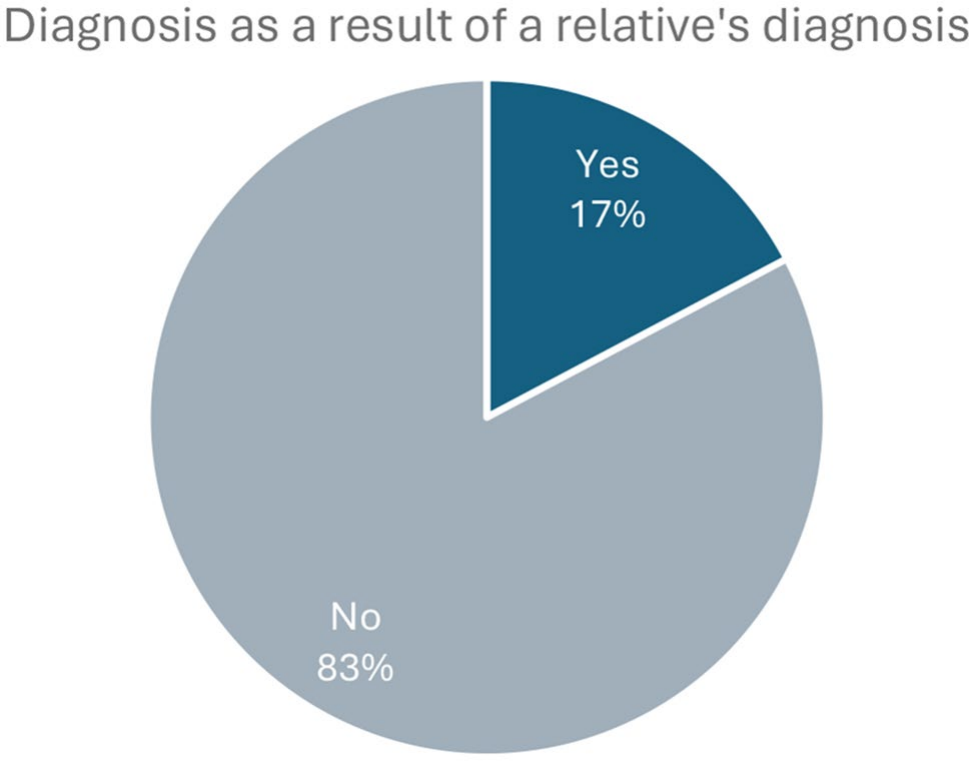
Responses to the survey question “Was your diagnosis as a result of a relative being diagnosed with Gaucher disease?”

Of the respondents, only a small minority were diagnosed as a result of a relative being diagnosed with Gaucher disease (Fig. 4).

Interestingly, the diagnostic experience of respondents from the same country varied greatly so it was not possible to draw any conclusions on the local experience or the healthcare system in each of the countries represented. Furthermore, the number of responders differed greatly between countries, preventing any regional insights being drawn out.

Patients reported seeing a wide range of specialists as part of their diagnostic journey, with nearly half of respondents seeing 3 or more (Table 1).

**Table 1:** The total number of specialists seen in the journey to diagnosis, as reported by respondents

Total number of specialists seen in the journey to diagnosis	Responses	
1	39%	48
2	15%	19
3 or more	46%	56

**Fig. 5:**
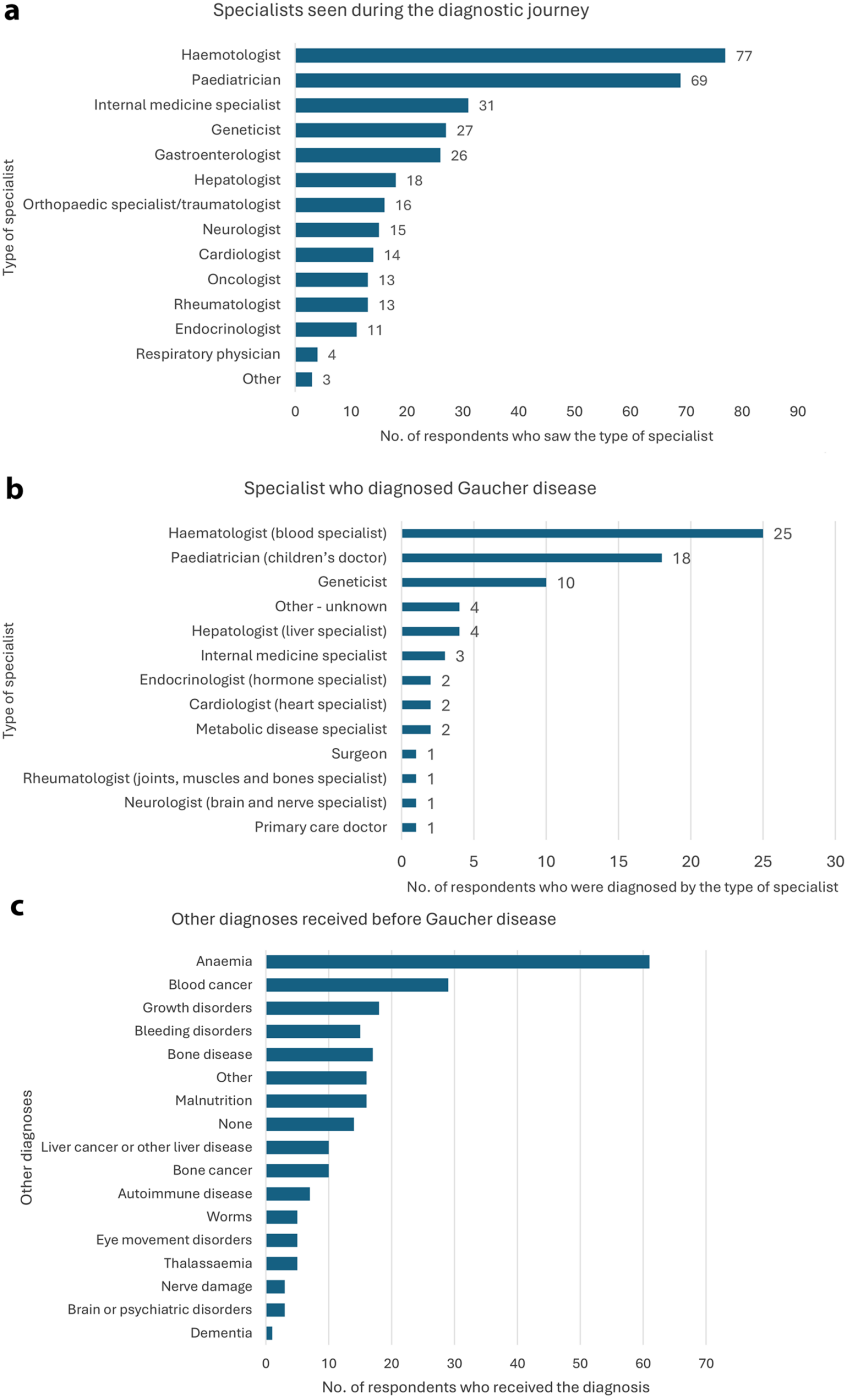
**a**. The range of specialists consulted by patients before a confirmed diagnosis of Gaucher disease. **b**. Diagnosis of Gaucher can be made by a range of specialists as shown in the above figure. **c**. Patients reported a wide range of conditions that were suggested or diagnosed prior to the final confirmation of Gaucher disease diagnosis

While there are many specialists who are commonly seen during the diagnostic journey due to the variable presenting symptoms characteristic of Gaucher disease with its different types (Fig. 5a), diagnosis is most commonly made by a haematologist or a paediatrician, followed by a geneticist (Fig. 5b). Patients are also likely to receive a diagnosis of other conditions before Gaucher disease, and these can be as diverse as anaemia, haematological malignancies or malnutrition (Fig. 5c).

People living with Gaucher and their caregivers reported a range of symptoms that confirmed Gaucher disease to their doctors. In many cases, respondents described the typical red flag symptoms of hepatosplenomegaly, anaemia, thrombocytopaenia and bone pathology. The latter symptoms were either described by patients differently or patients presented with other symptoms as growth delay or abnormal eye movement as shown in Table 2.

**Table 2:** Symptoms that supported a Gaucher disease diagnosis by physicians (NB. Using the lay terminology of the patients / caregivers)

Enlarged spleen (splenomegaly)
Extreme fatigue
Abdominal pain
Nose bleeds
Chronic anaemia
Severe & quick onset bone pain or when exercising
Severe unexplained bruising
Delay in growth / small for age
Large belly
Frequent vomiting
Abnormal eye movement

### Proposed causes of delay in diagnosis of Gaucher disease patients

Patients were asked to detail where in the diagnostic process any delays were experienced. While 23% of respondents did not experience any delays, there was a variety of reasons for delay given by the remaining respondents. The most commonly mentioned was “Lack of awareness of Gaucher disease and how it presents in different ways” (37%), and “Different specialists did not link the different symptoms together as having one cause (Gaucher disease)” (25%). Detailed responses from people living with Gaucher and their caregivers are shown in Table 3.

**Table 3:** Patient perceptions of reasons behind any delay in diagnosis of Gaucher disease. Other reasons cited included lack of testing, or slow and inaccurate test results

Reasons for delay in diagnosis as perceived by people living with Gaucher disease and their carers	No. of responses	% against total respondents
I did not experience any delays in my diagnosis	33	23%
Delays in seeing specialists	18	13%
Misdiagnosis e.g. time spent with the wrong physicians such as cancer specialists	33	23%
Lack of communication between specialists	23	16%
Different specialists did not link the different symptoms together as having one cause (Gaucher disease)	35	25%
Dismissive physicians who did not listen to you	26	18%
Lack of awareness of Gaucher disease and how it presents in different ways	52	37%
Seeing non-medical professionals for support (e.g. healers, religious leaders, homeopathic specialists)	4	3%
Other	17	12%

**Fig. 6:**
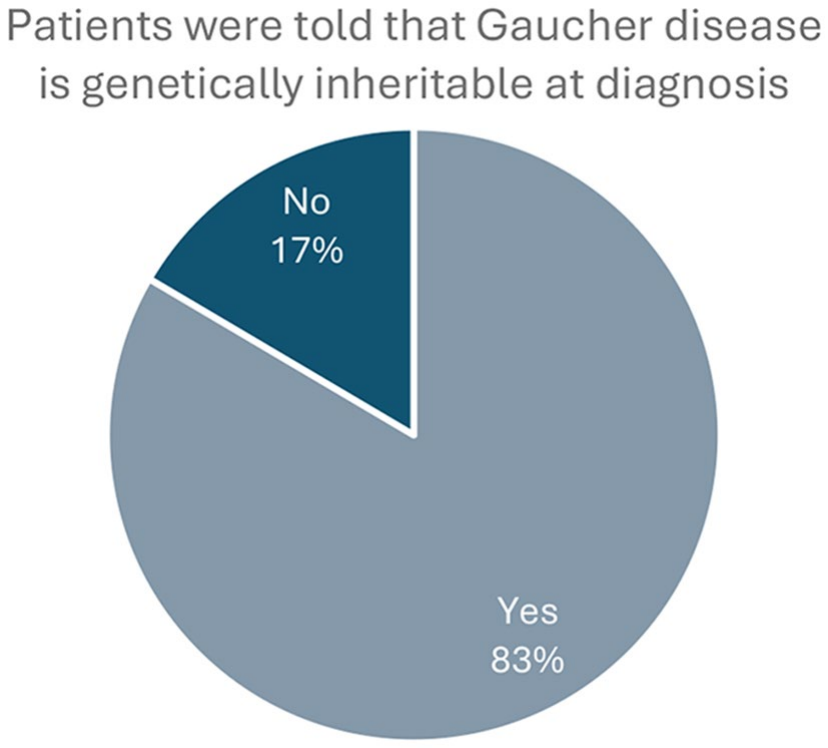
Responses to the survey question “When you were diagnosed with Gaucher disease, were you made aware that it is usually an inherited genetic disease?”

The respondents were also asked, based on their personal experience, how to shorten the time from presentation of first symptoms through to diagnosis with Gaucher disease. Increasing awareness and education of healthcare professionals and medical students, both on rare diseases in general and Gaucher disease specifically, were the primary suggestions. Almost half of respondents also called improved access to gene and enzyme testing (49%) and for an increase in the levels of newborn screening (44%) (Table 4). Access challenges extended beyond the tests themselves, with 37% calling for better access to specialist doctors. In addition, almost 1 in 5 (17%) of respondents were not made aware of the genetic nature of Gaucher disease at the time of their diagnosis (Fig. 6).

**Table 4:** Suggestions from surveyed people living with Gaucher disease on potential approaches to shorting the time to a confirmed diagnosis of Gaucher disease. Other proposed suggestions include improved access to testing, improved specialist care, and premarital / preconception genetic testing to identify gaucher carriers

How could time to diagnosis be shortened?	No. of responses	% against total respondents
Increase awareness of rare disease among healthcare professionals	89	63%
Increase awareness of Gaucher disease among healthcare professionals	80	56%
Increase awareness of rare disease and Gaucher disease among medical students	78	55%
Improve access to gene and enzyme testing	70	49%
Better access to specialist doctors	53	37%
Better patient education	32	23%
More accessible online education for healthcare professionals	28	20%
Increase use of newborn screening	63	44%
Other (please specify)	7	5%

### Communication process and multidisciplinary care of Gaucher disease

**Fig. 7:**
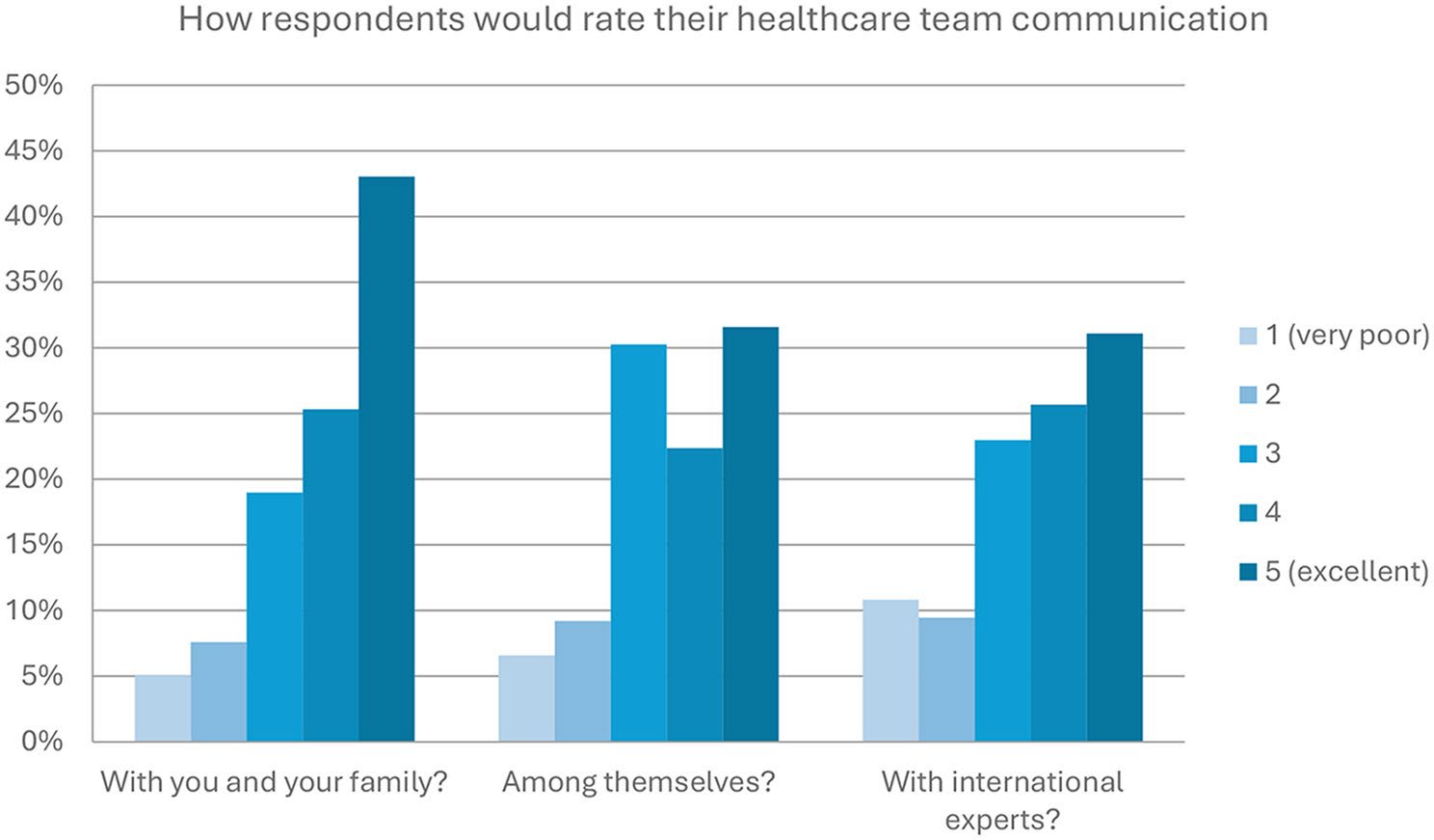
Participants in the survey were asked to rate the quality of communication of the healthcare practitioners currently involved in their care

Participants were asked to rate how they see the quality of communication around their current care as part of the survey. They were asked to share their view on how well the healthcare practitioners involved in their current care communicate: with themselves and their family; with the other healthcare practitioners involved in their care (for example, across multidisciplinary aspects of care); and finally, how their healthcare team engages with international experts. Overall, communication with the patient and family scored highest with 68% scoring 4 or 5 out of 5, followed by communication with international experts (57%), and just over half (54%) rated communication between the different healthcare practitioners involved in one person’s care as 4 or 5 out of 5 (see Fig. 7).

**Fig. 8:**
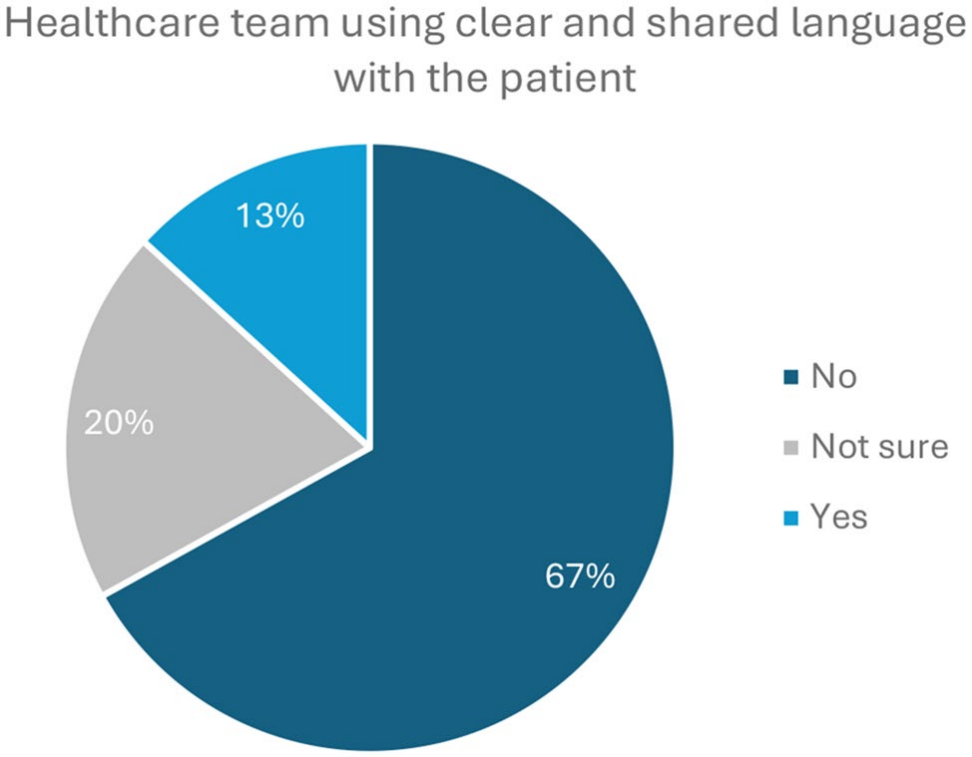
Participants reported that the terminology used by their healthcare team was clear and the same words were used in the majority of cases

At the end of the survey, people living with Gaucher disease and their caregivers were asked whether they find their physician uses different words to describe the condition that can make communication challenging. Interestingly, there was little experience of a mismatch between the physicians’ terminology and the lay understanding of the patients / caregivers (Fig. 8).

## Discussion

The aim of the survey was to highlight the ongoing challenges experienced by people living with Gaucher disease in achieving a confirmed diagnosis. It is well known that rare diseases are a diagnosis challenge by their very nature, meaning that patients often experience long and frustrating delays before a management plan and treatment can be put in place. Our survey explores the patient / caregiver perspective on the diagnosis of Gaucher aiming to identify touchpoints where improvements can be implemented.

While the working hypothesis for the survey was that diagnosis continues to be delayed, it was found that many patients were satisfied with the diagnostic process. Nonetheless, there remain areas where organisations like the IGA can provide support.

Overall, the survey showed that diagnostic delays remain a common feature for patients today, with 58% of respondents (excluding the 7% of those surveyed that did not comment on length of delay) experiencing a delay of more than a year. The reasons for delay were diverse but low awareness among physicians, lack of communication between different medical specialties and referrals to experts who are not familiar with Gaucher disease all contributed. The symptoms that triggered a confirmation of diagnosis appear to be the main “red flag” symptoms detailed in published guidelines. This suggests a need to raise awareness of these “red flags” among healthcare professionals – particularly those that are commonly seen by Gaucher patients in their journey but are not typically involved in an accurate diagnosis (e.g. internal medicine specialists and gastroenterologists). Interestingly, 17% of respondents were not made aware of the genetic nature of Gaucher disease at the time of diagnosis and therefore opportunities to diagnose the condition in relatives and future children would be missed.

When asked to suggest how the diagnostic journey could be accelerated, respondents most frequently indicated that increased awareness of rare diseases and Gaucher disease among healthcare professionals would be of value. Increased access to newborn screening and gene / enzyme testing were also highlighted as factors that could speed up diagnosis. The IGA, as part of their Global Gaucher Connect programme (GGCP) has carried out work to map access barriers to testing and specialist care [11] and these latest survey results highlight that this remains a prominent, global challenge.

The final section of our survey addressed issues around communication as a potential cause of diagnostic delays. Our survey shows that the patients felt that healthcare practitioners communicated well with the patient and their family, but less well with the extended healthcare team and with international experts in the field. We found that the mismatch in terminology used by patients and healthcare providers may be less than previously assumed, although the authors recognise that respondents approached by members of the IGA are likely to be well-informed and could be classed as ‘expert patients’ (well informed, active participants in their care) [12]. 

While some simple solutions can be implemented to ease short-term pressure and support communication between specialties at a local level, the infrastructure of health systems needs overall reform to ensure uninterrupted patient-centred care and a smoother and faster path to diagnosis. Patient-held records may be an alternative solution to alert different specialties to the broad range of seemingly unrelated symptoms, increasing the likelihood of recognising the connection between symptoms in different organ systems.

The authors are aware that this survey has shortcomings due to the limited number of respondents, the potential bias towards a well-informed patient population as well as a bias towards English- and Spanish-speaking respondents. A potential continuation of the research would be to provide further language versions of the survey to extend the reach to include greater geographic representation, thereby ensuring the broader relevance of these findings.

Beyond the reach of the survey, we also acknowledge limitations in the design of the survey which was based on working assumptions that were not as widely accepted as thought e.g. the assumption that communication between HCPs and patients is less than ideal and the assumption that diagnosis is often / always delayed.

## Conclusion and recommendations

One of the most common suggestions in our research from people living with Gaucher disease to shorten the time to diagnosis was to increase awareness and understanding of rare diseases including Gaucher disease among non-Gaucher-disease-specialist physicians and provision of educational support for healthcare teams.

The lack of uniform knowledge about the hereditary nature of Gaucher disease is surprising and should be focused on as a priority, with genetic counselling made part of the diagnostic process. Early identification of family members who may be affected through family pedigree analysis, as well as pre-marital / pre-conception testing and newborn screening, can all expedite diagnosis. Increased awareness, availability of and access to genetic testing will also alleviate the stress for patients and their families associated with the diagnostic journey.

Participants in the survey also highlighted that access to specialist physicians and to enzyme testing is limited. Increasing access through better awareness of these specialist services, ensuring that simple tests are readily available and improving funding for rare diseases will be welcomed by the community to ensure that all patients are able to access equitable care and treatment.

Based on the results of this survey, the IGA plans to review priorities and work with stakeholders in the Gaucher community to support Gaucher awareness programmes among non-Gaucher-disease-specialist physicians, particularly around the red flags of Gaucher disease and lobby for additional funding, testing and access to specialist care. Our primary recommendations for action informed by the survey results can be summarised as follows:

### Recommendation #1

Targeted efforts to raise awareness of Gaucher “red flags” among secondary care clinicians that are likely to see Gaucher patients on their journey but do not commonly diagnose, have the potential to significantly reduce time to diagnosis. Besides the common specialties that typically make Gaucher disease diagnoses (paediatricians, haematologists, geneticists), internal medicine specialists and gastroenterologists have been identified as prime targets for such an awareness campaign in our survey.

### Recommendation #2

Developing resources to support specialists who are typically involved in Gaucher diagnosis to explain genetic inheritance in lay-friendly terms to every person they diagnose, could help more family members be diagnosed at an earlier stage.

### Recommendation #3

Understanding the local experience of diagnosis through similar surveys of people living with Gaucher disease and their families would be instrumental in shaping country-specific interventions to improve the diagnostic journey.

## Supplementary Information

Below is the link to the electronic supplementary material.

Supplementary Material 1 Full survey for people living with Gaucher disease/caregivers

Supplementary Material 2 Sentiment analysis report

## Data Availability

All data generated or analysed during this study are included in this published article [and its supplementary information files].

## Abbreviations

IGA: International Gaucher Alliance
HCP: Healthcare professional
