# Supplementary material for: Exploring delayed diagnosis in Gaucher disease: insights from a community survey and potential solutions
Source: Orphanet J Rare Dis. 2026 Feb 20;21:125. doi: 10.1186/s13023-026-04209-5 (PMC13045069; doi:10.1186/s13023-026-04209-5)
Supplement: Supplementary file 1 — Supplementary Material 1 Full survey for people living with Gaucher disease/caregivers [file 13023_2026_4209_MOESM1_ESM.pdf]

perplexity

Analyze the sentiment of the survey responses in the uploaded Excel file. Provide a breakdown of positive, negative, and neutral sentiments, along with key insights and trends

Here's a sentiment analysis of the survey responses you provided, along with a breakdown, key insights, and trends:

Overall Sentiment Distribution:

Based on the responses, the overall sentiment is mixed, with a slightly negative leaning. Here's a rough estimate:

- **Negative:** 50% - Many responses express frustration, pain, and dissatisfaction with the diagnostic process, particularly regarding delays, invasive procedures, lack of knowledge among doctors, and misdiagnoses.
- **Neutral:** 30% - Some responses are factual accounts of the diagnostic process without strong positive or negative emotions. Others acknowledge improvements over time.
- **Positive:** 20% - These responses express gratitude for relatively quick diagnoses, satisfaction with current treatment, or relief at finally understanding their condition.

Key Insights and Trends:

1. **Diagnostic Delays:**
    - A significant number of respondents reported long delays in getting a correct diagnosis, sometimes spanning years.
    - These delays often led to unnecessary complications and emotional distress.
    - Several respondents mentioned that doctors were not initially aware of Gaucher disease, contributing to the delays.
2. **Invasive Procedures:**
    - Many respondents described painful and invasive procedures like bone marrow biopsies, liver biopsies, and splenectomies as part of the diagnostic process.
    - Some felt that these procedures were unnecessary, especially when less invasive options were available.
3. **Lack of Awareness/Knowledge:**
    - A recurring theme is the lack of awareness and knowledge about Gaucher disease among healthcare professionals.

    - This lack of knowledge often led to misdiagnoses or delayed diagnoses.
4. **Emotional Impact:**
    - The diagnostic process had a significant emotional impact on patients and their families.
    - Many respondents described feelings of fear, anxiety, frustration, and helplessness.
5. **Improvements Over Time:**
    - Some respondents who were diagnosed more recently noted that the process has improved compared to the past.
    - Increased awareness and better diagnostic tools have led to quicker diagnoses in some cases.
6. **International Disparities:**
    - Several responses highlighted disparities in access to diagnosis and treatment based on geographic location.
    - Some respondents had to travel to other countries to get a diagnosis or receive treatment.
7. **Impact on Quality of Life:**
    - Some respondents mentioned the impact of Gaucher disease on their quality of life, including challenges in personal life and career.

Specific Examples:

- **Negative:** "It took us 2 years to discover that is Gaucher disease... and in a meantime my sister stayed with out spleen."
- **Neutral:** "I was diagnosed when I was a few months old and therefore I don't remember it"
- **Positive:** "Once diagnosed things moved quickly."

Recommendations:

- **Increase Awareness:** Efforts should be made to raise awareness of Gaucher disease among healthcare professionals, especially general practitioners and specialists who may encounter patients with related symptoms.
- **Improve Diagnostic Protocols:** Streamline diagnostic protocols to reduce delays and minimize the need for invasive procedures.
- **Provide Support:** Offer emotional support and resources to patients and families throughout the diagnostic process.
- **Address Disparities:** Work to address disparities in access to diagnosis and treatment across different geographic regions.
- **Promote Research:** Continue to promote research to improve understanding of Gaucher disease and develop more effective treatments.

Disclaimer: This sentiment analysis is based on the provided text and is intended to provide a general overview of the survey responses. A more detailed analysis may be required to draw definitive conclusions.
