# Supplementary material for: Exploring delayed diagnosis in Gaucher disease: insights from a community survey and potential solutions
Source: Orphanet J Rare Dis. 2026 Feb 20;21:125. doi: 10.1186/s13023-026-04209-5 (PMC13045069; doi:10.1186/s13023-026-04209-5)
Supplement: Supplementary file 2 — Supplementary Material 2 Sentiment analysis report [file 13023_2026_4209_MOESM2_ESM.pdf]

# SURVEY FOR PEOPLE LIVING WITH GAUCHER DISEASE AND THEIR CAREGIVERS

**Thank you for participating in this International Gaucher Alliance (IGA) survey aimed at improving understanding of the diagnosis of Gaucher disease. Your insights will be used by the IGA to inform recommendations to speed up the diagnosis of this rare condition and to develop common terminology to improve communication between people living with Gaucher disease, types 1, 2 and 3 and their healthcare practitioners.**

**Purpose and Use of Data**
**This survey is designed to gather information about your experience of the diagnosis of Gaucher disease. The data collected will be used to improve services for people living with Gaucher disease and may be shared with Gaucher disease patient organisations and third-party researchers, but your identity will not be known.**

**Data Protection and Legal Compliance**
**We take all necessary steps to protect your personal information and comply with applicable laws. Your information will be kept confidential and used solely for the purposes of this survey.**

**No Professional Relationship Formed**
**Participating in this survey does not establish a doctor-patient relationship or any other professional relationship between you and the survey conductors. The survey is designed solely for research purposes and does not constitute medical advice or diagnosis.**

**Third-Party Links**
**This survey may include links to external websites. We are not responsible for the content or privacy practices of these linked sites. Please review the privacy policies of any external sites before providing personal information.**

**Anonymity and Confidentiality**
**Your responses to this survey will be kept anonymous and confidential. We will not associate your answers with your personal information unless you explicitly consent to such association. Your feedback will contribute to a general analysis and will not be used to identify individual participants.**

**Consent and Participation**
**Your participation in this survey is voluntary. You may withdraw from the survey at any time without any consequences. By continuing with the survey, you consent to the use of your data as described above.**
**By proceeding with the survey, you acknowledge that you have read, understood, and agree to these terms.**

## SECTION 1: TELL US ABOUT YOURSELF

* 1. Which country do you live in?

* 2. Are you...?

- ◯ A person living with Gaucher disease
- ◯ A carer for a person living with Gaucher disease
- ◯ A parent of a child with Gaucher disease
- ◯ Other (please specify)

* 3. If responding to this survey as a carer or parent, please answer the questions from the patient's perspective.

- ☐ I understand

* 4. What age (in years) were you when you first experienced symptoms of Gaucher disease?

- ◯ 17 or younger
- ◯ 18-24
- ◯ 25-34
- ◯ 35-44
- ◯ 45-54
- ◯ 55-64
- ◯ 65 or older

* 5. How many years was it before your diagnosis of Gaucher disease was confirmed?

* 6. Which type of Gaucher disease do you have?

- ◯ Gaucher type 1
- ◯ Gaucher type 2
- ◯ Gaucher type 3
- ◯ Not sure

7. Overall, how satisfied are you with the process of diagnosis of Gaucher disease?

Very dissatisfied (unhappy) — Very satisfied (happy)

8. Please explain your answer to the previous question

## Gaucher Disease Diagnosis Survey

### SECTION 2: DIAGNOSTIC JOURNEY

* 9. Was your diagnosis as a result of a relative being diagnosed with Gaucher disease?

- ○ No
- ○ Yes

## Gaucher Disease Diagnosis Survey

10. How was your Gaucher disease discovered? What were your first symptoms of Gaucher disease? Please describe them in your own words, and include any medical terminology used by healthcare professionals if known

## Gaucher Disease Diagnosis Survey

* 11. Which consultants did you see during your diagnostic journey? Please select all that apply.

- ☐ Cardiologist (heart specialist)
- ☐ Endocrinologist (hormone specialist)
- ☐ Gastroenterologist (stomach specialist)
- ☐ Geneticist
- ☐ Haematologist (blood specialist)
- ☐ Hepatologist (liver specialist)
- ☐ Internal medicine specialist
- ☐ Neurologist (brain and nerve specialist)
- ☐ Oncologist (cancer specialist)
- ☐ Orthopaedic specialist (bone specialist) / Traumatologist
- ☐ Paediatrician (children’s doctor)
- ☐ Respiratory physician (lungs and breathing)
- ☐ Rheumatologist (specialist in joints and muscles)
- ☐ Other (please specify)

* 12. Which type of specialist made the diagnosis of Gaucher disease?

- ◯ Cardiologist (heart specialist)
- ◯ Endocrinologist (hormone specialist)
- ◯ Gastroenterologist (stomach specialist)
- ◯ Geneticist
- ◯ Haematologist (blood specialist)
- ◯ Hepatologist (liver specialist)
- ◯ Internal medicine specialist
- ◯ Neurologist (brain and nerve specialist)
- ◯ Oncologist (cancer specialist)
- ◯ Orthopaedic specialist (bone specialist) / Traumatologist
- ◯ Paediatrician (children’s doctor)
- ◯ Respiratory physician (lungs and breathing)
- ◯ Rheumatologist (specialist in joints and muscles)
- ◯ Other (please specify)

* 13. Before you were diagnosed with Gaucher disease, what other conditions were suggested / diagnosed? Please select all that apply.

- ☐ Anaemia
- ☐ Autoimmune disease
- ☐ Bone cancer
- ☐ Bleeding disorders
- ☐ Brain or psychiatric disorders
- ☐ Growth disorders
- ☐ Blood cancer i.e. leukaemia or lymphoma
- ☐ Dementia
- ☐ Liver cancer or other liver disease
- ☐ Eye movement disorders
- ☐ Nerve damage
- ☐ Bone disease
- ☐ Thalassaemia
- ☐ Worms in the stomach
- ☐ Malnutrition
- ☐ None
- ☐ Other (please specify)

* 14. What were the symptoms that confirmed Gaucher disease to your doctors? Please describe the symptoms in your own words and also provide the medical terminology of your doctor, if possible.

* 15. If you experienced any delays in your diagnosis, what were these? Please select all that apply.

- [ ] Delays in seeing specialists
- [ ] Misdiagnosis e.g. time spent with the wrong physicians such as cancer specialists
- [ ] Lack of communication between specialists
- [ ] Different specialists did not link the different symptoms together as having one cause (Gaucher disease)
- [ ] Dismissive physicians who did not listen to you
- [ ] Lack of awareness of Gaucher disease and how it presents in different ways
- [ ] Seeing non-medical professionals for support (e.g. healers, religious leaders, homeopathic specialists)
- [ ] I did not experience any delays in my diagnosis
- [ ] Other (please specify)

* 16. In your opinion, how could the time from first symptoms to diagnosis for Gaucher patients be shortened? Please select all that apply

- [ ] Increase awareness of rare disease among healthcare professionals
- [ ] Increase awareness of Gaucher disease among healthcare professionals
- [ ] Increase awareness of rare disease and Gaucher disease among medical students
- [ ] Improve access to gene and enzyme testing
- [ ] Better access to specialist doctors
- [ ] Better patient education
- [ ] More accessible online education for healthcare professionals
- [ ] Increase use of newborn screening
- [ ] Other (please specify)

* 17. When you were diagnosed with Gaucher disease, were you made aware that it is usually an inherited genetic disease?

- [ ] Yes
- [ ] No
- [ ] If yes, were you offered genetic counselling around the time of your diagnosis? Please share anything about the information you received

## SECTION 3: COMMUNICATION

* 18. On a scale of 1 (very poor) to 5 (excellent), how would you rate the quality of communication from the healthcare practitioners involved in your care today?

| | 1 (very poor) | 2 | 3 | 4 | 5 (excellent) |
|---|---|---|---|---|---|
| With you and your family? | ○ | ○ | ○ | ○ | ○ |
| Among themselves? | ○ | ○ | ○ | ○ | ○ |
| With international experts? | ○ | ○ | ○ | ○ | ○ |

Please share any additional information here

* 19. Do you find that you and your physician use different words to describe your condition?

- Yes
- No
- Not sure

If yes, please provide any examples and, if you can, tell us which type of specialist this refers to

20. As an expert patient, we would be grateful if you could you describe the symptoms you know in the list below in simple terms for other patients

Fatigue:

Splenomegaly:

Hepatomegaly:

Thrombocytopenia:

Anaemia:

Bone pain:

Osteopenia:

Abnormal eye movement:

Developmental delay:

Seizures:

Poor coordination:

Spasticity/jerking movements:

21. **Conclusion**

Thank you for your time and insights. Your responses will contribute to improving the diagnosis of Gaucher disease.

**OPTIONAL:** Please provide your name, email address and telephone number ONLY if you are happy to be contacted in future about this research project, including focus groups.

| | |
|---|---|
| Name: | |
| Email: | |
| Telephone number (including country code, eg +44 for UK): | |
